# A cystic fibrosis gene editing approach that is on target

**DOI:** 10.1016/j.omtn.2024.102197

**Published:** 2024-05-08

**Authors:** Joseph J. Porter, John D. Lueck

**Affiliations:** 1 Department of Pharmacology and Physiology, University of Rochester School of Medicine and Dentistry, Rochester, NY 14642, USA; 2 Department of Neurology, University of Rochester School of Medicine and Dentistry, Rochester, NY 14642, USA; 3 Center for RNA Biology, University of Rochester School of Medicine and Dentistry, Rochester, NY 14642, USA

## Main text

**Figure 1:**
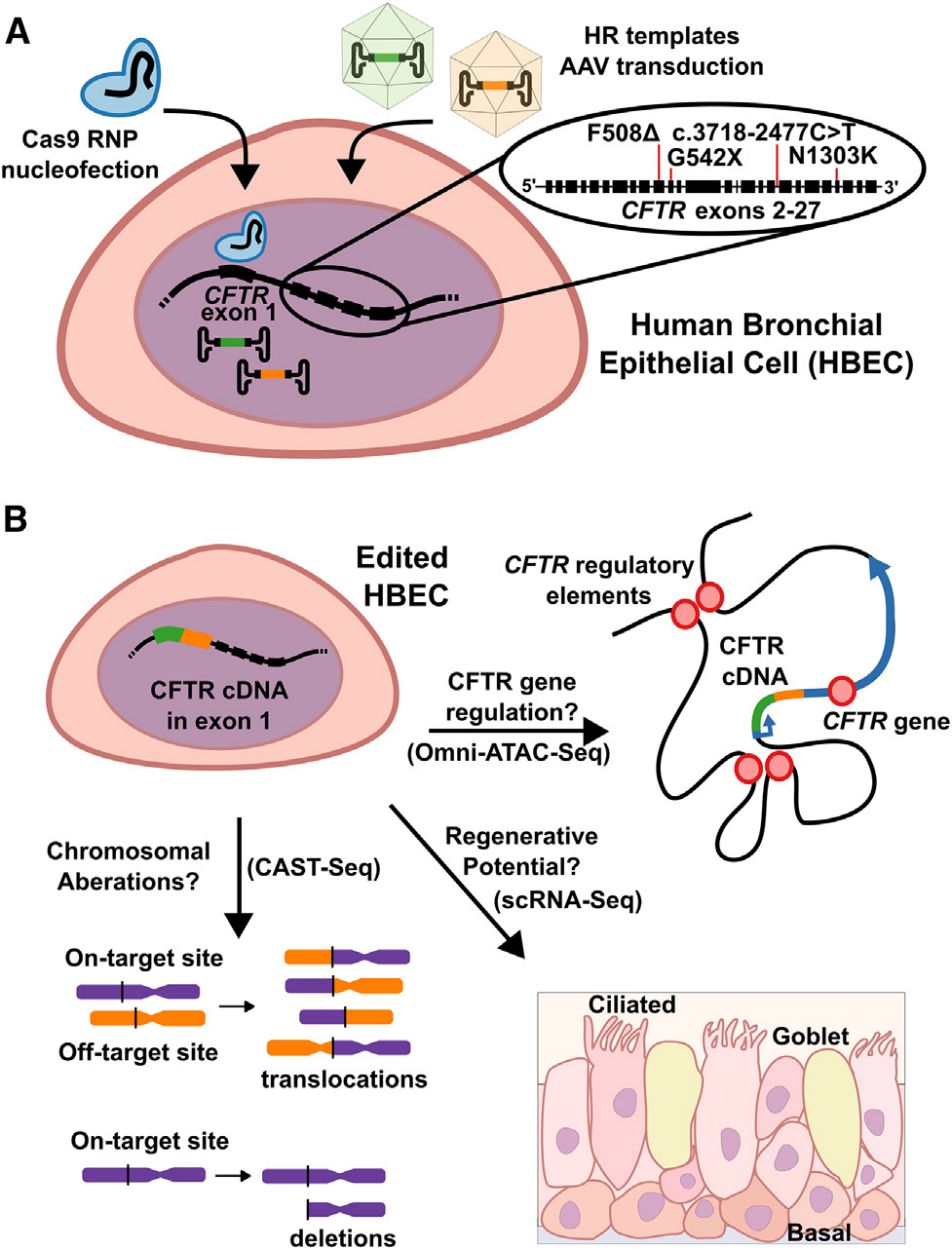
Tackling outstanding questions related to the “universal strategy” for *CFTR* correction (A) The universal strategy for correction of the *CFTR* gene in human bronchial epithelial cells (HBECs) is accomplished by nucleofection of a Cas9 ribonucleoprotein complex (RNP), composed of a high-fidelity Cas9 and single-guide RNA (sgRNA) modified with 2′-*O*-methyl-3′-phosphorthioate modifications on the last 3 bases on either end, targeting the first exon of the *CFTR* locus. The template for homologous recombination (HR) is supplied through transduction of AAV6 vectors each containing one-half of the HR template for the CFTR cDNA expression cassette (orange and green AAV genomic cargo). The inset shows some of the most common *CFTR* variants resulting in cystic fibrosis (CF). As *CFTR* exon 1 is upstream of all these variants, in principle, this strategy provides a path toward mutation-agnostic correction. (B) When assaying corrected HBECs, chromosomal aberrations analysis by single targeted linker-mediated PCR sequencing (CAST-seq), a recent approach for quantitative assessment of chromosomal aberration, revealed aberrant sequences amounting to only ∼1% of alleles. Here, two different chromosomes are represented in orange and purple, with possible translocations and deletions shown. The assay for transposase-accessible chromatin with sequencing (ATAC-seq) of corrected *CFTR* alleles containing CFTR cDNA revealed minimal changes in open-chromatin state as compared to wild-type *CFTR*. The *CFTR* genomic locus is shown with regulatory elements denoted as red circles. Single-cell RNA sequencing (scRNA-seq) revealed that edited HBECs were able to differentiate into cell populations similar to unedited control basal cells.

Cystic fibrosis (CF) is a recessive monogenic disorder resulting from any of the hundreds of mutations to the CF transmembrane regulator (*CFTR*) gene. While successful development and FDA approval of CFTR modulators has been a major therapeutic advance for many people with CF (pwCF), ∼8% of pwCF harbor CFTR mutations refractory to modulator treatment.^1^ Successful development of a mutation agnostic approach to CF correction promises a therapeutic for all pwCF. In this issue of *Molecular Therapy Nucleic Acids*, Vaidyanathan et al. demonstrated the safety of inserting *CFTR* cDNA with a transcription termination sequence in exon 1 of the *CFTR* locus (Figure 1A) as a “universal strategy” for rescue of >99% of CF-causing *CFTR* variants (Figure 1A, inset).^2^ The end result of the genetic manipulation is expression of normal CFTR mRNA under the control of the endogenous cellular transcription machinery. Analysis of this CRISPR-Cas9-based therapeutic gene complementation strategy presented here indicates that the insertion of *CFTR* cDNA by double-strand break and homologous recombination (HR) does not pose significant concerns regarding adverse genomic rearrangements or loss of regenerative potential of edited human basal epithelial cells (Figure 1B). Genome editing approaches offer the potential of a durable therapy for CF and other genetic disorders by correcting the underlying genetic lesions leading to disease. The outstanding concern related to the safety of this approach has been addressed here, with the results bolstering support for this and related large cDNA insertion approaches. However, questions remain as to how detrimental even the small amount (∼1%) of chromosomal aberrations resulting from genome editing are with this approach, which could become a roadblock in translation to the clinic. For CF, hurdles remain in implementing this approach, whether it is successful implantation of genome-edited cells/tissue if editing is performed *ex vivo* or successful targeting and delivery of the editing materials to airway epithelial basal cells *in vivo*.

CFTR modulators have undoubtedly been a major advancement in the quality of care for pwCF; however, modulators must be administered daily, are costly, only partially restore function, and do not cover the full spectrum of *CFTR* mutations.^3^ *CFTR* mutations can be grouped by mechanism for loss of CFTR channel activity, with class I mutations, including nonsense and splicing mutations, interfering with production of CFTR protein. As little to no CFTR protein is made, modulators alone will not work, and treatment of class I mutations will likely require approaches that directly modulate the aberrant CFTR mRNA transcript or replace the mutant CFTR mRNA or DNA sequence entirely. A multitude of approaches are being pursued, some of which include the development of small molecules targeting the ribosome, translation termination, or nonsense-mediated decay machinery; antisense oligonucleotides (ASOs; RNA-like short sequences that can bind and modulate their target RNA) for correction of splicing defects; RNA modification (targeted pseudouridylation) and nonsense suppressor tRNAs for seamless rescue of nonsense mutations; CFTR mRNA delivery via lipid nanoparticles; integration of *CFTR* cDNA by transposase; and delivery of *CFTR* cDNA by therapeutic adeno-associated viral (AAV) and lentiviral (LV) vectors.^1^^,^^4^ While ASO, AAV, and LV approaches have shown enough preclinical promise to begin clinical trials, these approaches may face a significant delivery hurdle to reach affected lung tissue, as they must transit through a thick mucus barrier and may require re-dosing, potentially leading to adverse or neutralizing immune responses. Further, many of these approaches are only applicable for subsets of *CFTR* mutations. Harnessing CRISPR-Cas9 genome editing for a universal strategy to insertion of *CFTR* cDNA provides a route to durable rescue of CFTR protein in a mutation-agnostic manner.

A platform consisting of Cas9, 2′-*O*-methyl-3′-phosphorthioate-modified single-guide RNA (MS-sgRNA), and a correction template for HR delivered by AAV6 was previously shown by the authors to effectively edit upper airway bronchial cells (UABCs) isolated from patients with CF.^5^ The *ex vivo* approach used in this study offers a potential alternative to *in vivo* gene therapy, as it avoids the thick mucus, immune reactions against Cas9, and toxic inflammatory environment present in patients that have been found to be barriers to efficient gene transfer *in vivo*. As a first step toward transplanting *ex vivo* gene-corrected UABCs into patients, UABCs were embedded into decellularized porcine small intestinal submucosa (pSIS) membranes, which are already in clinical use for several indications including sinonasal repair. Edited UABCs extracted from pSIS were found to differentiate similarly to wild-type UABCs when grown at the air-liquid interface (ALI) in culture, indicating that corrected epithelial basal cells should maintain their potential for differentiation when implanted in patients. Vaidyanthan and co-authors followed up this work with another publication outlining the universal strategy, a CF-variant-agnostic approach using a similar Cas9/MS-sgRNA platform with AAV6 HR donors cleverly designed with each containing one-half of a CFTR cDNA and tCD19 positive selection cassette targeted to exon 1/intron 1 of the *CFTR* locus (Figure 1A).^3^ While the results shown in these previous works provide a solid preclinical foundation, regulatory agencies have clearly stated the requirement for genome editing technologies to provide a preclinical assessment of specificity.^6^

The safety of the universal strategy of inserting a promoter-less *CFTR* cDNA expression cassette in exon 1 of the *CFTR* locus was thoroughly and carefully investigated here using chromosomal aberrations analysis by single targeted linker-mediated PCR sequencing (CAST-seq) to quantify chromosomal aberrations resulting from Cas9/MS-sgRNA delivery, Omni-assay for transposase-accessible chromatin with sequencing (ATAC-seq) to determine the impact of inserting the *CFTR* cDNA into the *CFTR* locus on the chromatin state of the *CFTR* gene, and single-cell RNA sequencing (scRNA-seq) to quantitatively profile the ability of corrected CF basal cells to properly differentiate into lung epithelial cell types (Figure 1B). To perform CAST-seq, UABCs from four donors were edited with Cas9/MS-sgRNA, and the isolated genomic DNA was sequenced for all four donors in two sequencing orientations to quantify aberrant events such as large insertions or deletions (indels) and translocations. The most frequent post-editing chromosomal aberration was large indels >50 bp in length in the CFTR locus, which occurred in only ∼1% of alleles in UABCs from all four donors. Of note, because the identified indels were located in the non-functioning CFTR being targeted for therapeutic complementation, any effect from these indels should be minimal. The next most frequently observed aberration, a translocation with chromosome 5 at a known off-target site,^3^ was observed in as few as 0.001%–0.01% of alleles. Less frequently observed was translocations within chromosome 7 (*CFTR* located on chromosome 7), which were present in ≤0.001% alleles but were not reproducible between donors. Human bronchial epithelial cells (HBECs) from 5 donors with CF were edited using the universal strategy, enriched for the tCD19 tag, and differentiated in ALI cultures. Differentiated cells maintained tCD19 expression, and CFTR channel function in the epithelial sheets was quantified by short-circuit current Ussing chamber measurements, with edited samples and non-CF samples showing similar levels of CFTR channel function. When Omni-ATAC-seq was used to quantify the chromatin state of the differentiated epithelial cells, no significant changes in the open-chromatin profile of the *CFTR* locus in response to gene editing using the universal strategy were noted, with extragenic enhancers at −44 and −35 kb remaining unchanged. Further, edited cells in ALI cultures from three of the donors were analyzed by scRNA-seq to determine the cell types present in the cultures. Indeed, the edited HBECs showed differentiation into the expected cell types, broadly consistent with the cell types of unedited healthy donors reported here and in other studies.

Genome editing strategies offer the promise of durable correction of disease-causing DNA mutations for CF along with many other inherited monogenic disorders. The results shown here indicate that the universal strategy to genomically correct CF along with related approaches can be on target and therefore safe. While no changes to the open-chromatin state or differentiated cell types were seen post-editing, the ∼1% of chromosomal aberrations noted is worth further consideration. One may expect that not all chromosomal aberrations are created equal and that the ∼1% noted here may be safe, while another editing platform or targeting other sites may lead to different results. The *ex vivo* correction of human epithelial basal cells obviates many of the challenges associated with *in vivo* genome editing; however, implantation of airway epithelial tissues and bioengineering functional lungs from scaffolds are open areas of research with challenges ahead.^7^ Further, because CF is a multisystem disease, targeting other affected cell types using this platform is of significant interest. Regardless of the challenges ahead for translation into the clinic, this study has set a benchmark for the implementation of therapeutic CFTR gene editing and the study of potential outcomes.
